# Controlled size reduction of rare earth doped nanoparticles for optical quantum technologies

**DOI:** 10.1039/c8ra07246a

**Published:** 2018-11-05

**Authors:** Shuping Liu, Diana Serrano, Alexandre Fossati, Alexandre Tallaire, Alban Ferrier, Philippe Goldner

**Affiliations:** Chimie ParisTech, PSL University, CNRS, Institut de Recherche de Chimie Paris F-75005 Paris France shuping.liu@chimieparistech.psl.eu philippe.goldner@chimieparistech.psl.eu; Sorbonne Université, Faculté des Sciences et Ingénierie UFR 933 F-75005 Paris France

## Abstract

Rare earth doped nanoparticles with sub-wavelength size can be coupled to optical micro- or nano-cavities to enable efficient single ion readout and control, a key requirement for quantum processors and high-fidelity single-ion quantum memories. However, producing small nanoparticles with good dispersion and exploitable optical coherence properties, another key aspect for these applications, is highly challenging by most synthesis and nano-fabrication methods. We report here on the wet chemical etching of Eu^3+^:Y_2_O_3_ nanoparticles and demonstrate that a controlled size reduction down to 150 nm, well below the wavelength of interest, 580 nm, can be achieved. The etching mechanism is found to proceed by reaction with grain boundaries and isolated grains, based on obtained particles size, morphology and polycrystalline structure. Furthermore, this method allows maintaining long optical coherence lifetimes (*T*_2_): the 12.5 μs and 9.3 μs values obtained for 430 nm initial particles and 150 nm etched particles respectively, revealing a broadening of only 10 kHz after etching. These values are the longest *T*_2_ values reported for any nanoparticles, opening the way to new rare-earth based nanoscale quantum technologies.

## Abstract 2

Chemical etching is a promising way to synthesize RE:Y_2_O_3_ nanoparticles with controlled size and long coherence lifetimes, opening the way to optical micro/nano-cavities coupling and efficient nanoscale quantum memories and processors.

## Graphical abstract

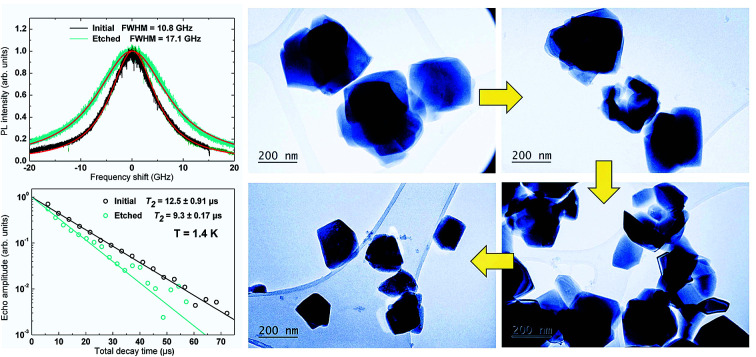

## 1 Introduction

Trivalent rare earths (REs) doped into a host material are attractive for multiple photonics and optoelectronics applications since they can present sharp absorption and emission lines, high quantum efficiency and no photobleaching.^1–4^ Recently, since the development of rare-earth doped nanoparticles, they are intensively investigated for bio-imaging,^5,6^ photodynamic therapy (PDT),^6^ lighting^7^ and security.^8^ In the last years, rare-earth doped crystals have also raised a strong interest in the field of optical quantum technologies, with a focus on quantum information processing and communication.^9–12^ Because of the shielding of 4f electrons by the outer filled 5s and 5p electron shells, the optical transitions of RE ions can exhibit very narrow optical and spin homogeneous linewidths (*Γ*_h_) at cryogenic temperatures, or equivalently, long optical and spin coherence lifetimes *T*_2_ = (π*Γ*_h_)^−1^. *T*_2_ corresponds to the lifetime of the superposition, *i.e.* quantum states that can be created in RE ions and are therefore of utmost importance in quantum technologies applications. Ions of interest in this field include Yb^3+^,^13^ Eu^3+^,^14^ Er^3+^,^15^ Nd^3+^,^16^ Pr^3+^ ^17^ and Tm^3+^ ^18^ doped into crystals such as Y_2_SiO_5_,^13–17^ YAG^18^ or YVO_4_.^19^ The longest optical coherence lifetime in a solid state system, *T*_2_ = 4.4 ms, was obtained in Er^3+^:Y_2_SiO_5_,^20^ and several hours of spin coherence lifetime have been reported in Eu^3+^:Y_2_SiO_5_.^21^ These attractive properties, unique in the solid state, allow RE doped crystals to be used as quantum light-matter interfaces, as shown in recent reports on quantum memories and optical to microwave transducers.^22–25^

Despite the striking demonstrations reported in macroscopic crystals, RE nanoparticles offer key advantages. In particular, nanoscale systems can be placed in a high-quality-factor optical micro- or nano-cavities to take advantage of a stronger light-matter coupling. This can lead to efficient single ion readout,^26,27^ and in the long term, it could give rise to scalable quantum processors and high fidelity memories for quantum networks.^19,28,29^

Nonetheless, obtaining long optical and/or spin coherence lifetimes in nano-materials like nano-diamonds^30^ or RE nano-crystals,^31^ is an outstanding challenge. This is because quantum states are extremely sensitive to even small time-dependent perturbations like fluctuating magnetic fields produced by impurities or defects carrying electron spins. At the nanoscale, such perturbing centres can form on particles surfaces or be introduced by precursors during the synthesis, which ultimately results in reduced coherence lifetimes. Very high quality materials are therefore needed to reach *T*_2_ values comparable to bulk samples. In this sense, Eu^3+^doped Y_2_O_3_ is a particularly interesting candidate: in contrast to other RE materials,^27,32–34^ Eu^3+^doped Y_2_O_3_ nanoparticles presenting remarkable optical and spin coherence properties, as well as narrow size distribution and bulk-like optical absorption and emission features can be obtained by chemical synthesis.^35–37^ Our previous studies on Eu^3+^:Y_2_O_3_ nanoparticles synthesized by homogeneous precipitation demonstrated an optical homogeneous linewidth of 45 kHz (*T*_2_ = 7 μs) for the Eu^3+^: ^7^F_0_ → ^5^D_0_ transition at 580 nm in 400 nm-diameter particles.^36^ Spin homogeneous linewidth down to 40 Hz (*T*_2_ = 8.1 ms) has also been recently reported.^37^ These results are comparable to values observed in some Eu^3+^:Y_2_O_3_ bulk crystals and transparent ceramics.^31,38–41^ Still, optical micro-cavities require low scattering losses in order to reach high quality factor and large cavity-ion coupling. Particles significantly smaller than the optical wavelength, 580 nm in the case of Eu^3+^, are therefore necessary, as the scattering losses scale as the sixth power of the particle size.^28^

Here, we propose wet chemical etching as a new approach to obtain well-dispersed and sub-wavelength RE doped Y_2_O_3_ nanoparticles with narrow homogeneous linewidths. Although the homogeneous precipitation method allows for morphology and size distribution control, the Y_2_O_3_ phase forms after calcination of the precipitated yttrium carbonate precursors (Y(OH)CO_3_·*x*H_2_O, YOC). Former investigations demonstrated that high calcination temperature (∼1200 °C) is required to cure defects, achieve sufficient crystalline quality and ensure good optical performance.^35,36^ However, this also leads to an increase in aggregation and sintering of the particles when their size is decreased. Our approach to prevent this consists in synthesising high-quality and well-dispersed Y_2_O_3_ particles with average size in the 400–500 nm range, and then applying chemical etching to achieve a controlled size reduction. This could allow obtaining particles with appropriate size with respect to the optical micro- or nano-cavity targeted quality factor, while preserving the initial particles optical properties. The etching mechanism and the optical coherence performance at 580 nm of Eu^3+^:Y_2_O_3_ etched particles were analyzed and are here discussed. Homogeneous linewidth broadening as low as 10 kHz was measured for 150 nm etched nanoparticles starting from 430 nm initial nanoparticles. The corresponding coherence lifetimes, 9.3 μs and 12.5 μs, are the longest ever reported for any nanoparticles. The results suggest that chemical etching is a promising way to obtain RE doped particles suitable for nanoscale quantum hardware architectures.

## 2 Materials and methods

The initial 0.3 at% Eu^3+^:Y_2_O_3_ nanoparticles were synthesized by homogeneous precipitation with a calcination temperature of 1200 °C. The detailed technological route has been described in previous work.^35^ The etching agent used in the study was glacial acetic acid (CH_3_COOH, original concentration of 100 wt%, with density 1.05 g cm^−3^). Acetic acid has been previously used as etchant in semiconductor manufacturing processes, with concentrations varying from 20 wt% to 68 wt%.^42^ The chemical etching experiments were carried out by mixing Eu^3+^:Y_2_O_3_ nanoparticles with fresh acid solutions (50 wt% prepared with deionized water). To ensure homogeneous temperature in the acid solution, etching was done under water-bath, with continuous magnetic agitation. After chemical etching, the nanoparticles were collected by centrifugation and washed several times with deionized water and absolute ethanol to remove the byproducts. The final etched powders were obtained after drying at 80 °C for 24 h. A post microwave treatment under oxygen plasma was performed to remove possible impurities. In order to clarify the effect of various etching conditions on the structures of the nanoparticles, etching time from 1 to 5 h, water-bath temperature from 40 to 70 °C and acetic acid concentration from 40 to 70 wt% were investigated. No dependence on the acetic acid content was found, while the effect of etching time and temperature is later discussed in the manuscript.

The morphology of initial and etched particles was observed by scanning electron microscopy (SEM, Zeiss Leo1530) and transmission electron microscopy (TEM, JEOL-JEM-100CXI) operating at 100 kV. The particle size distributions were calculated with Image J software based on at least 300 nanoparticles from different SEM images. X-ray diffraction (XRD) measurements were performed on a Panalytical XPert Pro diffractometer with an incident beam Ge monochromator. Crystallite or single grain sizes were determined from the FWHM of 4 different diffraction peaks by applying the Scherrer equation. Mass losses due to etching were derived from the concentration of yttrium in the etched solutions measured by inductively coupled plasma atomic emission spectrometry (ICP-AES, ThermalFisher icp 6000).^43,44^

Inhomogeneous and homogeneous linewidths were measured on several initial and etched particles with different average particle size after etching. The samples in form of powders were placed in a helium bath cryostat (Janis SVT-200) and maintained in a copper holder between two glass plates. The detection was carried out by collecting light scattered through the sample as explained in previous works.^31,36^ Inhomogeneous linewidths (*Γ*_inh_) were recorded, for the Eu^3+^: ^7^F_0_ → ^5^D_0_ transition, at approximately 10 K by fluorescence excitation using a CW dye ring laser (Sirah Matisse DS, 200 kHz linewidth) pumped by a Coherent Verdi G10 laser. A long-pass filter (600 nm cut-off wavelength) was placed in front of the detector (APD Thorlabs 110 A/M) to reject the excitation light. Homogeneous linewidths (*Γ*_h_) were determined from coherence lifetimes (*T*_2_), the latter measured by two pulse photon echo experiments at the center of the ^7^F_0_ → ^5^D_0_ (580.88 nm in vacuum) transition at 1.4 K. The length of the exciting and rephasing pulses in the sequence was 1 and 1.5 μs respectively.

## 3 Results and discussion

### 3.1 Structural characterizations and etching mechanism

**Fig. 1:**
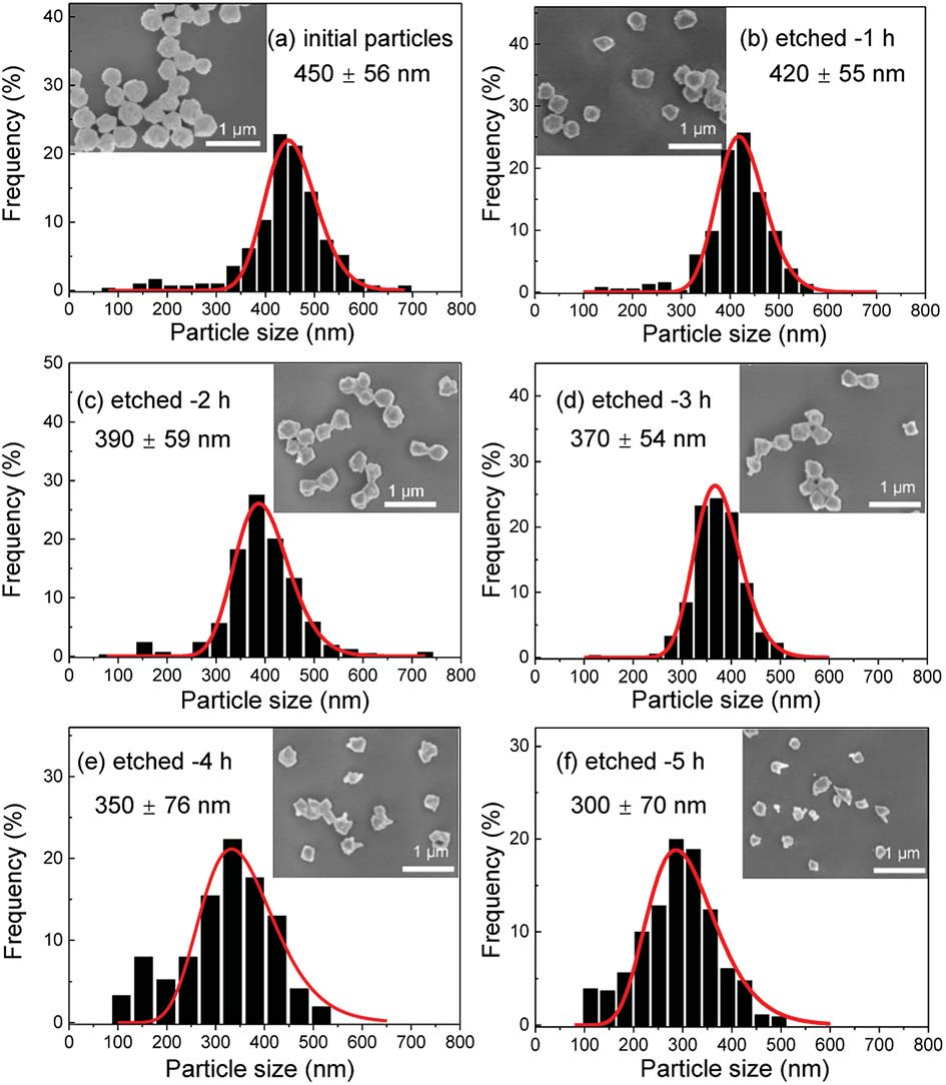
Scanning electron microscopy (SEM) micrographs and size distributions of the initial and etched Eu^3+^:Y_2_O_3_ nanoparticles for etching times increasing from 1 to 5 hours (etching temperature *T* = 50 °C).

Several series of Eu^3+^doped Y_2_O_3_ nanoparticles were synthesized by homogenous precipitation, calcined at 1200 °C, and subsequently etched in acetic acid solutions of 50 wt% acid content (8.53 mol L^−1^). A weak acid (p*K*_a_ = 4.76 at 25 °C) was chosen for low etching rates, allowing control over the size reduction process. Fig. 1 shows the evolution of the particle size distribution and morphology as a function of etching time. The initial Eu^3+^:Y_2_O_3_ nanoparticles exhibit spherical, well-dispersed morphology and particle size of 450 ± 56 nm according to log-normal fit. As observed, when etching time increases from 1 to 5 hours, the nanoparticles turn to be gradually smaller and their shape changes, as evidenced by the appearance of sharp edges and facets. It can be noted that the size distribution of the etched particles stays comparable to that of the initial particles, slightly broadening after 4 hours etching (Fig. 1d and e).

**Fig. 2:**
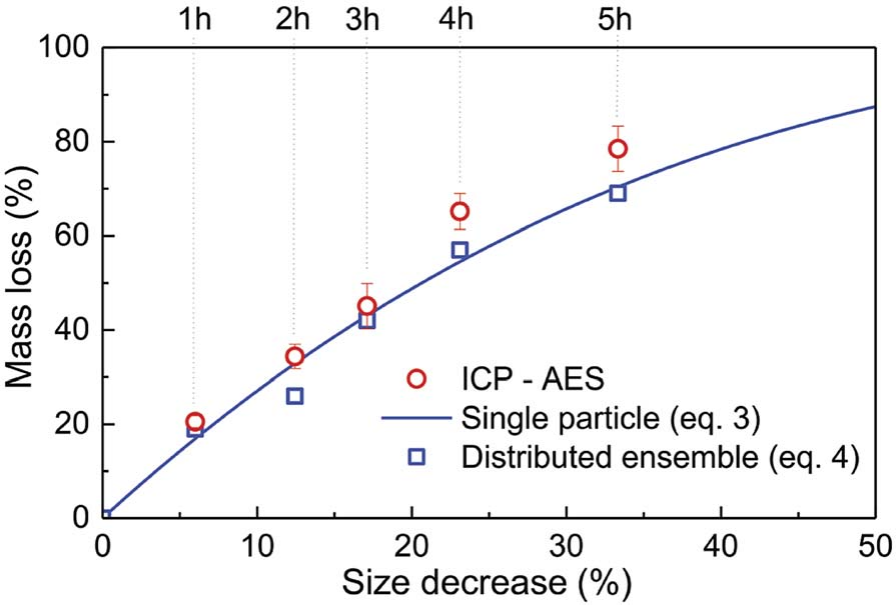
Relative mass loss due to etching as a function of the relative particle size decrease, (*r*_0_ − *r*_e_)/*r*_0_, with *r*_0_ = 225 nm (initial particles) and *r*_e_ the average radius of the etched particles after different etching times, *e.g. r*_0_ = 150 nm for *t* = 5 h (Fig. 1). The experimental ICP-AES results (red dots) are compared to the mass loss expected from the size reduction model (eqn (3) and (4)) for a single average particle (line) and an ensemble of 1000 particles distributed in size as in Fig. 1 (blue squares).

Inductively coupled plasma atomic emission spectroscopy (ICP-AES) was used to determine the mass loss due to etching by measuring the yttrium element concentration in the acid solution after removing the etched particles. In Fig. 2, the ICP-AES results are compared to the expected mass loss based on the progressive size reduction observed in Fig. 1. This simple model assumes that the nanoparticles are perfectly spherical and that the total number of particles remains the same along the etching process. The mass of a single particle is then given by:1
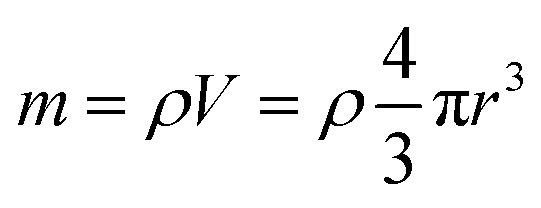
in which *V* in the particle volume, *r* the particle radius and *ρ* the Y_2_O_3_ density. After an etching time *t*, the radius decreases from *r*_0_ to a specific value *r*_e_, and the mass being etched for each particle, Δ*m*, can be expressed as:2
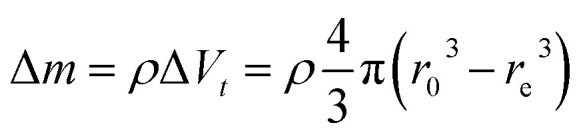


Then, the relative mass loss per particle just depends on the initial and final particle radii as:3
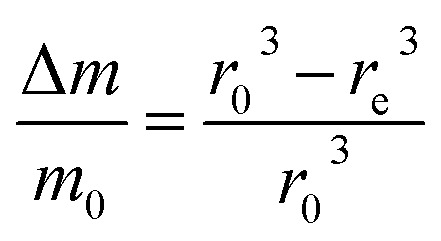


For an ensemble of particles with total mass 
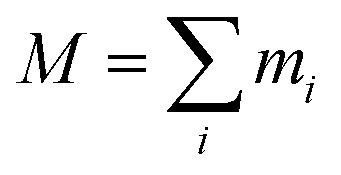
 and a distribution of initial and final radii *r*_0,*i*_ and *r*_e,*i*_, eqn (3) becomes:4
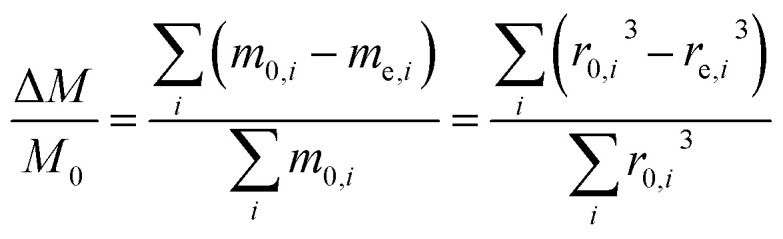


**Fig. 3:**
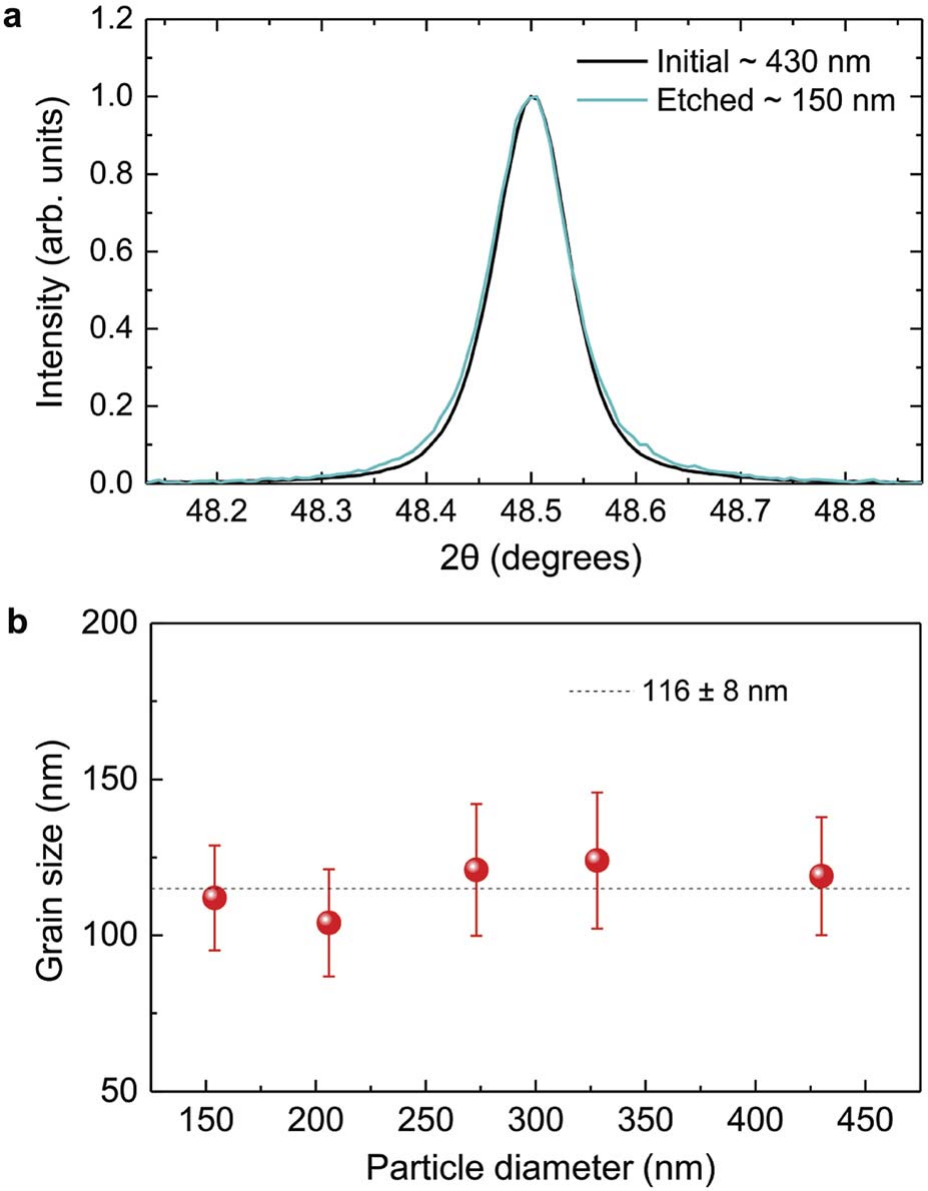
XRD investigation. (a) Normalized diffraction peak for initial and etched particles with average sizes of 430 and 150 nm respectively. (b) Grain size as a function of particle size. The dotted line is a guide to the eye.

**Fig. 4:**
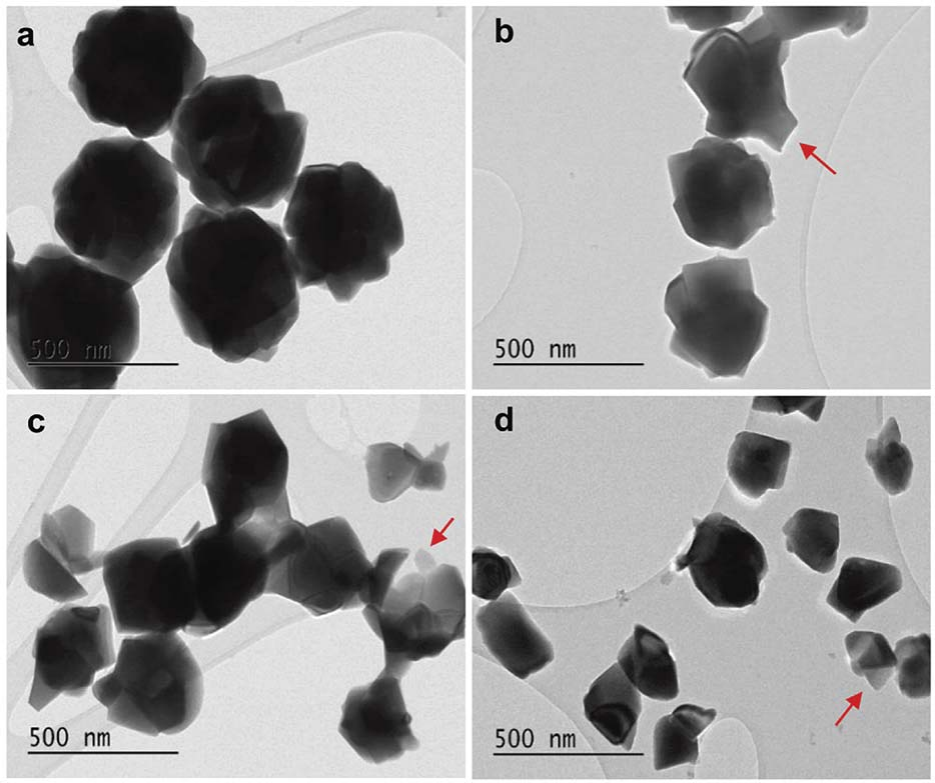
TEM images of (a) initial 450 nm-diameter particles. Etched particles with final average particle size of (b) 400 nm, (c) 340 nm and (d) 200 nm obtained by adjusting etching time and temperature (see Fig. 5). Red arrows: single crystalline grains nearly detached from particles during etching.

As displayed in Fig. 2, the mass loss trend measured by ICP-AES is rather well described by eqn (3) and (4), some deviation appearing for long etching times (>4 h). A closer insight into the etched particles inner structure nevertheless indicates that this model, just considering an isotropic and continuous volume reduction of the particles, does not provide an accurate description of the etching mechanism taking place here. Indeed, as the initial Eu^3+^:Y_2_O_3_ particles are polycrystalline, they are made of multiple crystalline grains with average sizes ranging from ≈100 to 120 nm. XRD investigations reveal that the etching process does not lead to a noticeable reduction of the grain size nor increases the grain size dispersion (Fig. 3), in disagreement with the continuous volume reduction hypothesis. To explain this observation, we propose that the acid preferentially reacts with grain boundaries, leading to the detachment of small crystal grains as the etching proceeds, as reported in polycrystalline silicon ultra thin films.^45^ This is supported by TEM images in which nearly detached grains are observed (Fig. 4, red arrows). The mass loss measured by ICP-AES and given in Fig. 2 is mainly attributed to detached grains, which are etched in a rather short time due to their small volume.

**Fig. 5:**
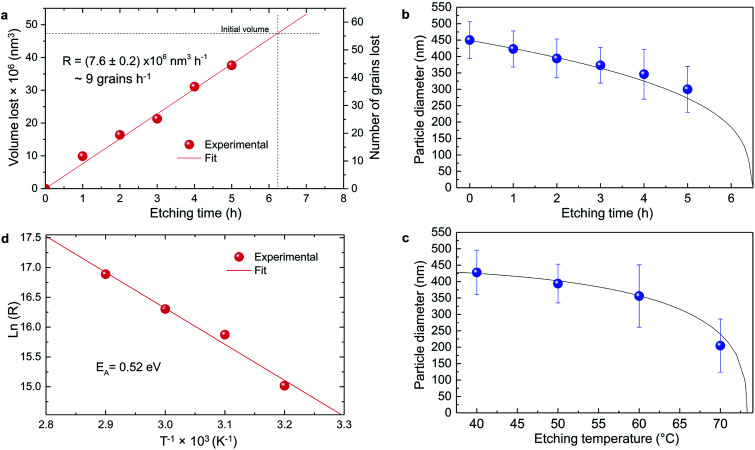
Particle size evolution with etching time and temperature. (a) Volume lost as a function of etching time and corresponding etching rate. (b) Particle size decrease as a function of etching time with *T* = 50 °C (dots) compared to the particle size evolution expected from a rate of 7.6 × 10^6^ nm^3^ h^−1^ (line). (c) Particle size decrease as a function of etching temperature with a fixed etching time of 2 h (dots) compared to the particle size evolution for an activation energy *E*_A_ = 0.52 eV (line). (d) ln(*R*) *versus T*^−1^ showing a linear dependence from which *E*_A_ can be estimated according to eqn (8).

Based on the previous conclusions, we redefined the size reduction model by taking into account the polycrystalline nature of the particles, with average grain diameter of 116 ± 8 nm (Fig. 3b). The mass of a single particle then becomes:5
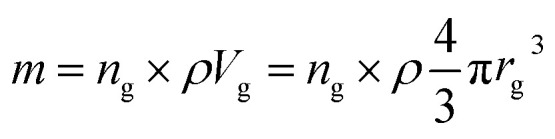
in which *V*_g_ is the volume of a single crystalline grain, *n*_g_ is the number of grains in the particle and *r*_g_ the grain radius. The relative mass loss per particle is then given by:6
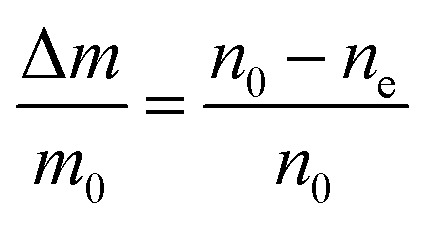
where *n*_0_ is the number of grains in the initial particle and *n*_e_ the number of grains in the final etched particle. From eqn (6), the number of grains which are lost (*n*_0_ − *n*_e_), *i.e.* detached and then fully etched, can be estimated as a function of etching time from the experimental mass loss measured by ICP-AES (Fig. 2). The result is shown in Fig. 5a. The etching rate appears linear, equal to ∼9 grains h^−1^, or equivalently, 7.6 × 10^6^ nm^3^ h^−1^ at *T* = 50 °C. This result is indeed consistent with several experimental evidences: at the rate found, the initial particles of ∼450 nm should be completely etched after approximately 6.5 hours (Fig. 5b). This was indeed observed since almost no particles could be collected after 6 hours etching. The etching rate found also implies that single crystalline grains are dissolved in less than 7 minutes, therefore contributing to the mass loss shortly after being isolated from the particles.

The temperature dependence of the etching rate, *R*(*T*), was also determined from the particle size decrease as a function of etching temperature for a fixed etching time *t*. Thus, *R*(*T*) was estimated as:7
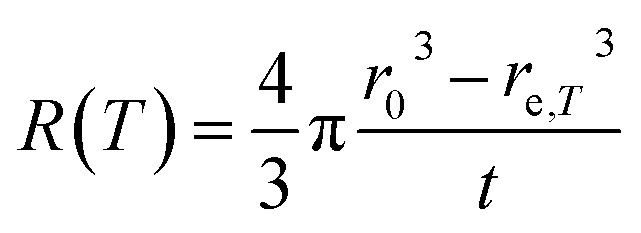
in which *t* was set equal to 2 hours, *r*_0_ = 225 nm and *r*_e,*T*_ is the average radius of the etched particles obtained with different etching temperatures (Fig. 5c). As displayed in Fig. 5d, *R*(*T*) follows the law:8
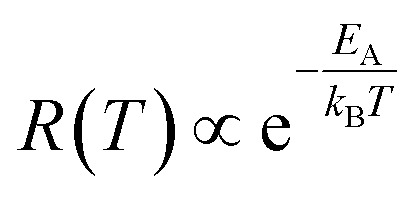
where *k*_B_ is the Boltzmann constant and *E*_A_ the activation energy, here found equal to 0.52 eV or 50.2 kJ mol^−1^ (Fig. 5d). This value corresponds to a surface-reaction-limited etching process.^46^

In conclusion, it is demonstrated that a controlled size reduction of the initial Eu^3+^:Y_2_O_3_ particles is possible by accurately tuning etching time and temperature. Particles smaller than 150 nm and even single crystalline particles could in principle be obtained by further optimization of both parameters.

### 3.2 High resolution and coherent spectroscopy

**Fig. 6:**
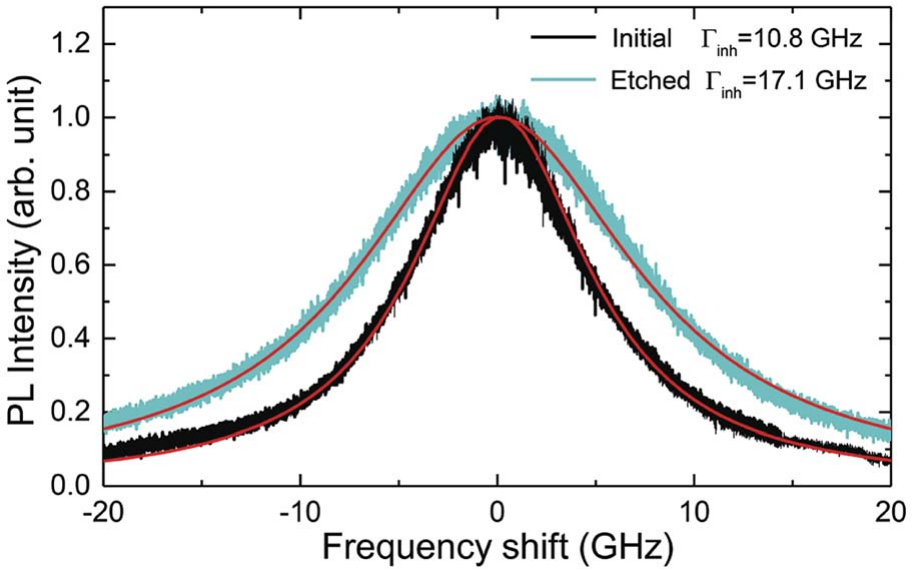
Inhomogeneous broadening (*Γ*_inh_) of the ^7^F_0_ → ^5^D_0_ optical transitions in initial (black) and etched (green) nanoparticles with average sizes of 450 and 200 nm respectively. Linewidths were estimated by Lorentzian fit.

The inhomogeneous broadening (*Γ*_inh_) of initial and etched particles was recorded at low temperature (∼10 K) by monitoring the Eu^3+ 5^D_0_ emissions while scanning a narrow-linewidth single frequency laser through the ^7^F_0_ → ^5^D_0_ transition (see Methods). The initial particles showed *Γ*_inh_ values around 11 GHz, as expected for particles annealed at 1200 °C.^35^ After etching, a clear broadening was observed in all measured samples, as in the example shown in Fig. 6. Both the initial and etched particles lineshapes could be described by Lorentzian functions. This denotes point defects as the source of inhomogeneous broadening.^47^ In non-etched particles, the inhomogeneous broadening is known to be dominated by the Eu^3+^ ions themselves, highlighting the low content of defects in these materials.^35^ Substitution of Y^3+^ by Eu^3+^ induces strain in the crystalline matrix and therefore broadening because of their different atomic radii: *r*_Eu_ = 0.950 Å, *r*_Y_ = 0.892 Å.^48^ Upon etching, strain is not expected to change in the crystalline grains since their size is not noticeably changed during the process, as discussed in the previous section. We therefore attribute the additional broadening observed in etched particles to an increase in point defects at the surface of the crystalline grains, in agreement with the Lorentzian lineshape of the transition.

**Fig. 7:**
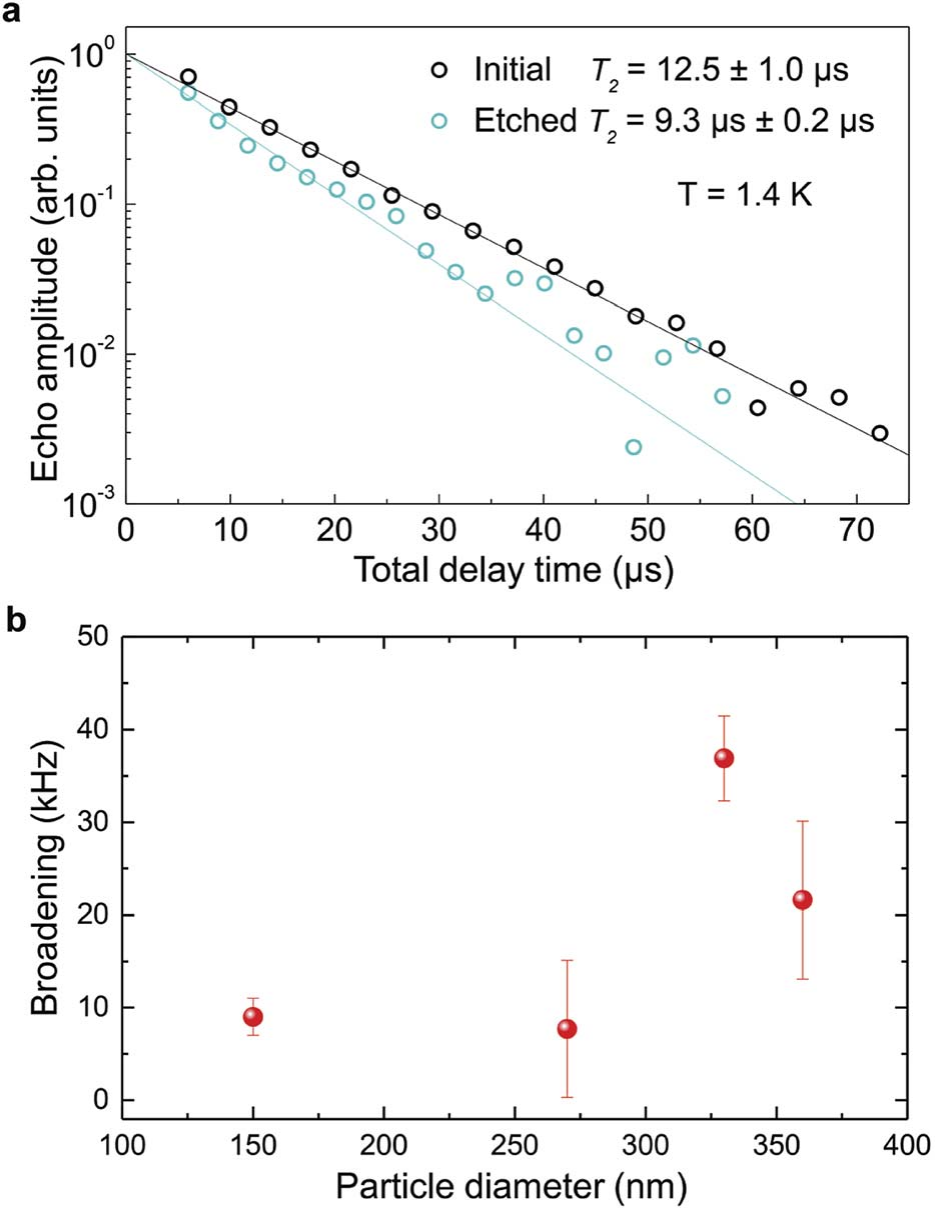
Optical coherence lifetimes and homogeneous broadening. (a) Photon echo decays for initial and etched nanoparticles at 1.4 K with average sizes of 430 and 150 nm respectively. *T*_2_ values were derived by single exponential fit to the decay data. (b) Additional broadening due to etching as a function of the particles size. Error bars represent ±1 standard deviation from several measurements.

In order to determine the impact of etching on the optical coherence of the particles, we measured optical coherence lifetimes (*T*_2_) in a series of etched nanoparticles. Starting from an initial size of 430 nm, the smallest particles had an average diameter of 150 nm after etching. Coherence lifetimes and homogeneous linewidths *Γ*_h_ = (π*T*_2_)^−1^ were obtained from two-pulse photon echo measurements^31,36^ at 1.4 ± 0.1 K (see Methods). As shown in Fig. 7a, they could be fitted by a single exponential, as previously observed in other nanocrystals.^31^ All measured homogeneous linewidths were below 100 kHz and the additional broadening attributed to etching (*Γ*_h,etched_ − *Γ*_h,initial_) did not show a clear dependence on particle size as displayed in Fig. 7b. In particular, the smallest particles homogeneous linewidth was broadened by only 10 kHz compared to the initial particles. In a previous study, we attributed the main contributions to Eu^3+^ homogeneous linewidth in nanoparticles to fluctuations due to residual disorder and surface charges.^36^ In all likelihood, the additional broadening induced by etching results from surface modifications (Fig. 4). Since the broadening is only weakly dependent on the particle size, it seems probable that it occurs at the crystalline grain level, whose size does not change with etching (Fig. 3). It could be explained by an increase of fluctuations related to disorder, which increases after etching as evidenced by *Γ*_inh_ measurements. Surface charges could also increase after etching due for example to the creation of dangling bonds. Further studies involving temperature dependence of *Γ*_h_ and 3-pulse echoes decays could help clarify this point.^36,40^

These results demonstrate that wet chemical etching can be very useful to reduce particle size while maintaining very narrow homogeneous linewidths. For the smallest particles, we are able to decrease particle size by 65%, from 430 nm to 150 nm, (96% reduction in volume), while obtaining *Γ*_h_ = 34.2 kHz, an unmatched value for any nanoparticle to the best of our knowledge. The significance of this linewidth can be illustrated by comparing it with the interactions used to implement quantum gates in rare earth doped crystals.^49^ Based on Eu^3+^ optical Stark coefficients, electric dipole–dipole interactions will induce frequency shifts equal to *Γ*_h_, *i.e.* 34.2 kHz, for ions separated by about 10 nm.^36^ In the 150 nm particles, the average distance between Eu^3+^ ions in the C_2_ site for 0.3% doped Y_2_O_3_ particles is 3.2 nm, whereas a single particle contains about 10^5^ Eu^3+^ ions. This suggests that a large fraction of ions could interact strongly enough to explore Eu^3+^–Eu^3+^ interactions and 2-qubit gates. Furthermore, a fibre micro-cavity containing a 150 nm particle could reach quality factors of a few 1000 s at 580 nm, an increase of nearly three orders of magnitude compared to 400 nm particles, which should be high enough to show enhanced light coupling with Eu^3+^ ions.^28^

## 4 Conclusion

In conclusion, we have found that chemical etching can be used for etching Eu^3+^:Y_2_O_3_ oxide polycrystalline nanoparticles at controlled rates. The particles size can be decreased from initial large particles in the 400–500 nm range to much smaller ones (*i.e.* 150 nm) with a narrow distribution, good dispersion and without obvious changes on the single crystallite size. Based on imaging and structural analysis we propose an etching mechanism that acetic acid tends to open the grain boundaries in the particles, detaching single crystalline grains that are quickly etched, and effectively reducing the size of the remaining particles. Furthermore, we demonstrate that chemical etching has a limited impact on optical performance of the nanoparticles, leading to homogeneous broadenings below 40 kHz for particles between 360 and 150 nm. Moreover, the coherence times of 12.5 μs (*Γ*_h_ = 25.5 kHz) for 430 nm initial particles and 9.3 μs (*Γ*_h_ = 34.2 kHz) for 150 nm etched nanoparticles observed at 1.4 K, are the longest optical coherence times ever reported for nanoparticles. Our results demonstrate that chemical etching is a promising way to synthesize RE:Y_2_O_3_ nanoparticles suitable for coupling with optical micro-cavities and with long coherence lifetimes, opening the way to efficient nanoscale quantum memories and processors.

## Conflicts of interest

There are no conflicts to declare.

## Supplementary Material

## References

[cit1] Weber M. J. , Handbook on the Physics and Chemistry of Rare Earths, Elsevier, 1979, vol. 4, pp. 275–315

[cit2] Digonnet M. J. F. , Rare-Earth-Doped Fiber Lasers and Amplifiers, Revised and Expanded, CRC Press, 2001

[cit3] Ronda C. R., Jüstel T., Nikol H. (1998). J. Alloys Compd..

[cit4] Rowan B. C., Wilson L. R., Richards B. S. (2008). IEEE J. Sel. Top. Quantum Electron..

[cit5] Bouzigues C., Gacoin T., Alexandrou A. (2011). ACS Nano.

[cit6] Abdesselem M., Schoeffel M., Maurin I., Ramodiharilafy R., Autret G., Clḿent O., Tharaux P.-L., Boilot J.-P., Gacoin T., Bouzigues C., Alexandrou A. (2014). ACS Nano.

[cit7] Sinha G., Patra A. (2009). Chem. Phys. Lett..

[cit8] Kim W. J., Nyk M., Prasad P. N. (2009). Nanotechnology.

[cit9] Thiel C. W., Böttger T., Cone R. L. (2011). J. Lumin..

[cit10] Tittel W., Afzelius M., Chaneliere T., Cone R. L., Kröll S., Moiseev S. A., Sellars M. (2010). Laser Photonics Rev..

[cit11] Goldner P., Ferrier A., Guillot-Noël O. (2015). Handb. Phys. Chem. Rare Earths.

[cit12] Kunkel N., Goldner P. (2018). Z. Anorg. Allg. Chem..

[cit13] Welinski S., Ferrier A., Afzelius M., Goldner P. (2016). Phys. Rev. B.

[cit14] Equall R. W., Sun Y., Cone R. L., Macfarlane R. M. (1994). Phys. Rev. Lett..

[cit15] Böttger T., Thiel C. W., Cone R. L., Sun Y. (2009). Phys. Rev. B.

[cit16] Usmani I., Afzelius M., de Riedmatten H., Gisin N. (2010). Nat. Commun..

[cit17] Equall R. W., Cone R. L., Macfarlane R. M. (1995). Phys. Rev. B.

[cit18] Louchet A., Habib J. S., Crozatier V., Lorgeré I., Goldfarb F., Bretenaker F., Le Gouët J.-L., Guillot-Noël O., Goldner P. (2007). Phys. Rev. B.

[cit19] Zhong T., Kindem J. M., Bartholomew J. G., Rochman J., Craiciu I., Miyazono E., Bettinelli M., Cavalli E., Verma V., Nam S. W., Marsili F., Shaw M. D., Beyer A. D., Faraon A. (2017). Science.

[cit20] Böttger T., Thiel C. W., Sun Y., Cone R. L. (2006). Phys. Rev. B.

[cit21] Zhong M., Hedges M. P., Ahlefeldt R. L., Bartholomew J. G., Beavan S. E., Wittig S. M., Longdell J. J., Sellars M. J. (2015). Nature.

[cit22] Bussières F., Clausen C., Tiranov A., Korzh B., Verma V. B., Nam S. W., Marsili F., Ferrier A., Goldner P., Herrmann H., Silberhorn C., Sohler W., Afzelius M., Gisin N. (2014). Nat. Photonics.

[cit23] Saglamyurek E., Jin J., Verma V. B., Shaw M. D., Marsili F., Nam S. W., Oblak D., Tittel W. (2015). Nat. Photonics.

[cit24] Maring N., Farrera P., Kutluer K., Mazzera M., Heinze G., de Riedmatten H. (2017). Nature.

[cit25] Williamson L. A., Chen Y.-H., Longdell J. J. (2014). Phys. Rev. Lett..

[cit26] Kolesov R., Xia K., Reuter R., Stöhr R., Zappe A., Meijer J., Hemmer P. R., Wrachtrup J. (2012). Nat. Commun..

[cit27] Utikal T., Eichhammer E., Petersen L., Renn A., Götzinger S., Sandoghdar V. (2014). Nat. Commun..

[cit28] Casabone B., Benedikter J., Hümmer T., Beck F., de Oliveira Lima K., Hänsch T. W., Ferrier A., Goldner P., de Riedmatten H., Hunger D. (2018). New J. Phys..

[cit29] Dibos A. , Raha M., Phenicie C. and Thompson J., arXiv:1711.10368v1, 2017

[cit30] Knowles H. S., Kara D. M., Atatüre M. (2014). Nat. Mater..

[cit31] Perrot A., Goldner P., Giaume D., Lovrić M., Andriamiadamanana C., Gonçalves R. R., Ferrier A. (2013). Phys. Rev. Lett..

[cit32] Eichhammer E., Utikal T., Götzinger S., Sandoghdar V. (2015). New J. Phys..

[cit33] Lutz T., Veissier L., Thiel C. W., Woodburn P. J. T., Cone R. L., Barclay P. E., Tittel W. (2016). Sci. Technol. Adv. Mater..

[cit34] Lutz T., Veissier L., Thiel C. W., Woodburn P. J. T., Cone R. L., Barclay P. E., Tittel W. (2017). J. Lumin..

[cit35] de Oliveira Lima K., Rocha Gonçalves R., Giaume D., Ferrier A., Goldner P. (2015). J. Lumin..

[cit36] Bartholomew J. G., Lima K. D., Ferrier A., Goldner P. (2017). Nano Lett..

[cit37] Serrano D., Karlsson J., Fossati A., Ferrier A., Goldner P. (2018). Nat. Commun..

[cit38] Flinn G. P., Jang K. W., Ganem J., Jones M. L., Meltzer R. S., Macfarlane R. M. (1994). Phys. Rev. B.

[cit39] Ferrier A., Thiel C. W., Tumino B., Ramirez M. O., Bausá L. E., Cone R. L., Ikesue A., Goldner P. (2013). Phys. Rev. B.

[cit40] Kunkel N., Bartholomew J., Welinski S., Ferrier A., Ikesue A., Goldner P. (2016). Phys. Rev. B.

[cit41] Karlsson J., Kunkel N., Ikesue A., Ferrier A., Goldner P. (2017). J. Phys.: Condens. Matter.

[cit42] Lee C.-Y., Chang C., Shih W.-P., Dai C.-L. (2010). Thin Solid Films.

[cit43] Atanassova D., Stefanova V., Russeva E. (1998). Talanta.

[cit44] Ferreira S. L. C., de Brito C. F., Dantas A. F., Lopo de Araujo N. M., Costa A. C. S. (1999). Talanta.

[cit45] Yoo S.-J. , Lee J. and Shin H., Digest of Papers Microprocesses and Nanotechnology 2000. 2000 International Microprocesses and Nanotechnology Conference (IEEE Cat. No.00EX387), 2000, pp. 248–249

[cit46] Tan S. , Boudreau R. and Reed M. L., Technical Digest. MEMS 2001. 14th IEEE International Conference on Micro Electro Mechanical Systems (Cat. No.01CH37090), 2001, pp. 139–142

[cit47] Stoneham A. M. (1969). Rev. Mod. Phys..

[cit48] Shannon R. D., Prewitt C. T. (1969). Acta Crystallogr., Sect. B: Struct. Crystallogr. Cryst. Chem..

[cit49] Walther A., Rippe L., Yan Y., Karlsson J., Serrano D., Nilsson A. N., Bengtsson S., Kröll S. (2015). Phys. Rev. A.

